# Susceptibility-weighted imaging at high-performance 0.5T magnetic resonance imaging system: Protocol considerations and experimental results

**DOI:** 10.3389/fnins.2022.999240

**Published:** 2022-10-12

**Authors:** Yueqi Qiu, Haoran Bai, Hao Chen, Yue Zhao, Hai Luo, Ziyue Wu, Zhiyong Zhang

**Affiliations:** ^1^School of Biomedical Engineering, Shanghai Jiao Tong University, Shanghai, China; ^2^Institute of Medical Robotics, Shanghai Jiao Tong University, Shanghai, China; ^3^Wuxi Marvel Stone Healthcare Co., Ltd., Wuxi, Jiangsu, China

**Keywords:** SWI, low-field, 0.5T MRI, protocol optimization, SNR improvement

## Abstract

The high-performance low-field magnetic resonance imaging (MRI) system, equipped with modern hardware and contemporary imaging capabilities, has garnered interest within the MRI community in recent years. It has also been proven to have unique advantages over high-field MRI in both physical and cost aspects. However, for susceptibility weighted imaging (SWI), the low signal-to-noise ratio and the long echo time inherent at low field hinder the SWI from being applied to clinical applications. This work optimized the imaging protocol to select suitable parameters such as the values of time of echo (TE), repetition time (TR), and the flip angle (FA) of the RF pulse according to the signal simulations for low-field SWI. To improve the signal-to-noise ratio (SNR) performance, averaging multi-echo magnitude images and BM4D phase denoising were proposed. A comparison of the SWI in 0.5T and 1.5T was carried out, demonstrating the capability to identify magnetic susceptibility differences between variable tissues, especially, the blood veins. This would open the possibility to extend SWI applications in the high-performance low field MRI.

## Introduction

Susceptibility-weighted imaging (SWI) is especially helpful in finding hemorrhage in traumatic brain injury and can also characterize cerebral microbleeds, occult low-flow vascular malformations, intracranial calcifications, neurodegenerative diseases (Haacke et al., 1997; Bartzokis et al., 2000; Halefoglu and Yousem, 2018). Phase information in SWI is used to emphasize the magnetic susceptibility differences of various compounds, such as deoxygenated blood, blood products, iron, and calcium, to provide a new source of contrast in MR (Haacke et al., 1995, 2004; Reichenbach et al., 1997). SWI shows better results in higher fields than in lower fields because the signal-to-noise ratio (SNR) increases with the field strength (Hoult et al., 1986; Sarracanie and Salameh, 2020; Runge and Heverhagen, 2022), and it is easier to get the optimum contrast for veins at high fields with much shorter echo times than that at low fields (Haacke et al., 2009). Since high-field MRI machines are expensive, heavy and need pricey annual maintenance costs, low field MRI machines gain an edge by their potential to offer reduced costs and reduced footprints translating into wider accessibility. Low field MRI can have key competitive advantages such as compact fringe fields, smaller-size, lighter-weight, more economic magnets, easier siting and installation requirements (Coffey et al., 2013; Stainsby et al., 2019, 2020; Wiens et al., 2020). Especially, Siemens has announced a low-field MRI, MAGNETOM Free. Max system, which combines low field strength (0.55T) with high-performance imaging technology and shows excellent image quality (Campbell-Washburn et al., 2019; Sheth et al., 2021). Meanwhile, Synaptive Medical has launched their recently FDA-approved 0.5T head-only MRI and discussed advantages in imaging at low-field compared with traditional high-field MRI (Marques et al., 2021; Jimeno et al., 2022).

Although lower main magnetic field strength magnets result in lower SNR, there are several unique physical advantages of low-field MRI compared to high-field MRI. First, T1 is shorter and T2 is longer under lower fields (Campbell-Washburn et al., 2019), which helps shorten TR as well as allows more SNR-efficient sampling. This would allow some imaging sequences to employ longer echo trains at low fields. Moreover, susceptibility-induced magnetic field gradients scale with *B*_0_, geometric distortion and blurring can be reduced due to the slower T2* decay at lower fields (Osmanodja et al., 2022). Low fields are also gaining more momentum because of the reduced SAR and the comfort level of patients (Stainsby et al., 2020; Bhat et al., 2021; Runge and Heverhagen, 2022; Wujciak, 2022). Furthermore, the susceptibility-based image artifacts caused by the changes in susceptibilities moving from air to tissue or by the metal implants are significantly reduced when the field strength decreases (Ejbjerg et al., 2005; Campbell-Washburn et al., 2019). Recently, studies of DWI and TOF MRI have shown the high-quality diagnostic values of low-field images (Hori et al., 2006; Runge and Heverhagen, 2022). With the help of a high-performance gradient system, pulmonary imaging of diffuse lung disease or focal pneumonia, which has not been evaluated by MRI in the past, shows good contrast and details at the low field (Hori et al., 2006). Though the quality of the SWI images improves substantially with increased field strength, with the new generation advanced low-field systems, it is possible to obtain SWI images with some initial results (Hori et al., 2021), while deserves further investigations to extend the SWI clinical applications at low fields.

In this study, we aim to make use of the physical advantages of the low field MRI to optimize imaging protocol to obtain SWI images as well as apply some denoising methods to improve the results. We optimized the values of time of echo (TE), repetition time (TR), and the flip angle (FA) of the RF pulse according to the decay law of the signal at 0.5T for SWI (Haacke et al., 2009). In order to improve the SNR performance of low-field images, principal component analysis and BM4D denoising method were adopted for denoising. In addition, we averaged the magnitude images obtained from multiple echo acquisitions to improve the SNR further. The SWI images acquired at 1.5T and 0.5T were compared for a better understanding of the SWI features at low fields.

## Methods

### Susceptibility effect considerations in 0.5T

Susceptibility-weighted imaging is generally acquired by a high-resolution 3D gradient-echo (GRE) sequence (Tong et al., 2008; Wu et al., 2009; Schweser et al., 2010). SWI makes use of the information of both phase and magnitude (Barnes and Haacke, 2009). To process the data in SWI, we applied phase unwrapping, and then used a high-pass filter to remove low-frequency fluctuations (Wang et al., 2000; Santhosh et al., 2009; Liu et al., 2015). A phase mask is next created to scale data from the filtered phase images over a 0–1 range (Wang et al., 2006; Wu et al., 2009). The magnitude image is digitally multiplied by this phase mask several times to highlight tissues with different susceptibilities. We can express the local phase difference as (Haacke et al., 2009).


(1) $$\begin{array}{c} △\,\varphi =-\gamma \,g\,△\,\chi \,B_{0}\,T\,E \end{array}$$

where *g* is a geometric factor, △χ is the difference of the local magnetic susceptibility of the tissue of interest from its surrounding, and *B*_0_ is the field strength.

According to the susceptibilities of biological tissues (Saini et al., 1988; Schenck, 1996), for example, deoxygenated blood has △χ= 0.45 ppm relative to surrounding tissue, iron containing proteins have an approximately △χ= 0.21 ppm with respect to cerebrospinal fluid (CSF), to make the susceptibility difference in phase of different tissues well-marked, △φ should be large enough. According to Eq. 1 and clinical experience of adequate △φ, the optimized TE at 0.5T becomes approximately 80–120 ms. However, long TE degrades the magnitude of the image, leading to low SNR. When considering whether the magnitude of the image is sufficient, we need to consider the attenuation formula of the signal.

### TR/TE/FA considerations for susceptibility weighted imaging in 0.5T

The magnitude signal intensity for the RF-spoiled gradient-echo sequence is given by


(2) $$\begin{array}{c} \rho_{m}\,\left(\theta \right)=\rho_{0}\,s\,i\,n\,\theta \,\exp\left(-\frac{T\,E}{T2{}^{*}}\right)\times \frac{\left[1-\exp\left(\frac{T\,R}{T\,1}\right)\right]}{\left[1-c\,o\,s\,\theta \,\exp\left(\frac{T\,R}{T\,1}\right)\right]} \end{array}$$

where ρ_0_ is the tissue spin density, TR is the repetition time, TE is the time of echo, T1 is the tissue longitudinal relaxation time constant, T2* is the corresponding transverse relaxation time constant and θ is the angle by which the magnetization is tipped.

Based on the T1 and T2* values of white matter (WM), gray matter (GM), and CSF at 0.5T and 1.5T from the literature as shown in Table 1 (Peters et al., 2007; Campbell-Washburn et al., 2019), we can optimize the image contrast that the GM/WM contrast is almost nonexistent. A slightly higher FA is chosen to partially suppress partially the CSF, making it slightly darker than GM or WM so that edema can appear hyperintense. For patients with tumors, this method is able to demonstrate not only the peritumoral edema but also the bleeding inside the tumor (Haacke et al., 2009).

**TABLE 1:** Parameter table for 0.5T and 1.5T (Peters et al., 2007; Campbell-Washburn et al., 2019).

Magnetic field (T)	Tissue	T1 (ms)	T2* (ms)
0.5	White matter	493	72
	Gray matter	717	86
	Cerebrospinal fluid	2,200	200
1.5	White matter	780	64
	Gray matter	920	83
	Cerebrospinal fluid	2,500	100

The signal-intensity behavior was first simulated as a function of flip angles for the given TR and TE, then parameters were chosen to satisfy the spin-density-like magnitude contrast requirements as well as achieve a relatively higher signal intensity. A certain length of TE also needs to be guaranteed to provide observable susceptibility differences between different tissues.

### Signal-to-noise ratio improvement for susceptibility weighted imaging in 0.5T

Due to the long TE requirement for revealing susceptibility effects in the 0.5T MRI, the SNR is inherently reduced. However, as the magnitude images from adjacent multi-echo acquisitions are similar due to the relative flat T2* decay at low fields, an average of the magnitude images obtained from a few echoes in the multi-echo acquisition can be used to improve the SNR. When selecting the echoes for averaging, the contrast of the images obtained from the echoes, should meet the similar-contrast requirements of SWI, where the GM/WM contrast is almost nonexistent and CSF is slightly darker. Moreover, due to excessive noise in some coils at low fields, a principal component analysis method is used to extract the data obtained by the first few principal components to achieve high quality images. In order to further improve the SWI results, the state-of-the-art the denoising algorithm BM4D can be used for denoising the phase map. BM4D is a four-dimensional block matching cooperative filtering, which has a good performance when it is used to remove Gaussian noise and Rician noise.

## Experimental

Experiments were performed at 0.5T and 1.5T systems on five healthy volunteers, who provided written informed consent, and followed procedures approved by our local Institutional Review Board. The high-performance 0.5T MRI system was ramped down from a customized 1.5T system developed by Wuxi Marvel Stone Healthcare while the 1.5T system used is the Siemens Aera 1.5T scanner (Siemens Healthineers, Erlangen, Germany). The GRE sequence parameters for 1.5T system were TR = 55 ms, TE = 40 ms, matrix size = 192 × 192 × 64, spatial resolution = 1.2 mm × 1.2 mm × 2 mm, flip angle = 20°. While the sequence parameters for the 0.5T system were TR = 90 ms, TE = 80 ms in single-echo sequence, TR = 90 ms, TE = 30, 40, 50, 60, 70, and 80 ms in multi-echo sequence, matrix size = 192 × 192 × 64, spatial resolution = 1.2 mm × 1.2 mm × 2 mm, flip angle = 20°.

## Results

### Susceptibility weighted imaging results from 0.5T

**FIGURE 1:**
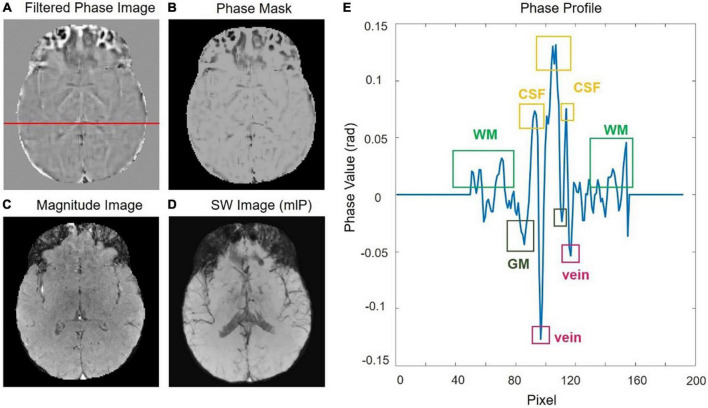
**(A)** The original phase image was filtered with a central filter size of 64 × 64 to get the HP-filtered phase image. **(B)** The phase mask. **(C)** The raw magnitude image. **(D)** Susceptibility weighted imaging (SWI) image by using a minimum intensity projection. **(E)** Phase profile from a row of the filtered-phase image [the red line in panel **(A)**].

The SWI processing is illustrated with a representative slice from the 0.5T *in vivo* acquisition as shown in Figure 1. Before getting the susceptibility weighted image, we need to get HP-filtered phase images, design the “phase mask” to enhance the contrast in the original magnitude and get the projection over 10 slices. A phase profile plot (Figure 1E) from the red line indicated in Figure 1A shows the phase difference of the tissues, such as WM, GM, CSF as well as veins, depending on their susceptibilities. All the results from five volunteers are listed in Supplementary Figure 1.

### TR/TE/FA optimization results

**FIGURE 2:**
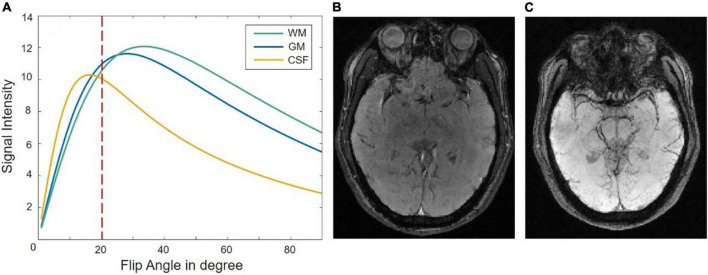
**(A)** A signal-intensity simulation plot as a function of flip angle for gray matter (GM), cerebrospinal fluid (CSF), and white matter (WM) at 0.5T with TE = 80 ms, TR = 90 ms. Panel **(B,C)** show the raw magnitude image and susceptibility weighted imaging (SWI) image when the flip angle is 20°.

A signal-intensity simulation based on Eq. 2 is given for WM, GM, and CSF at 0.5T when TE = 80 ms, TR = 90 ms in Figure 2. The intensity contrast of the different tissues of the raw image in Figure 2B fits the simulation curve of Figure 2A. The GM/WM contrast is almost nonexistent and CSF is suppressed and darker than the GM/WM. The magnitude image as shown in Figure 2B has a reasonable SNR of 47.1067 as calculated which the mean of the signal at the center (24 × 24) of the image divided by the variance of the signal at the four comers (24 × 24) of the image. The long TE at the low field could provide sufficient susceptibility effects between different tissues, the SWI image shows a pretty nice structure of veins as shown in Figure 2C.

### Denoised improvements

**FIGURE 3:**
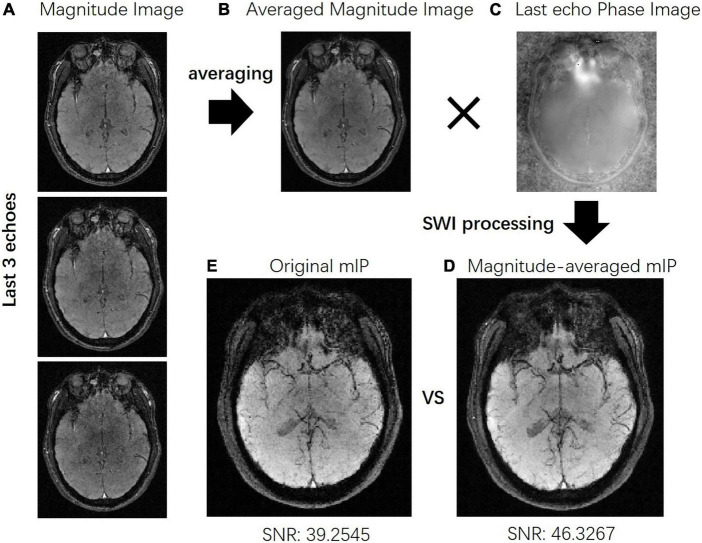
**(A)** The last three-echo magnitude images with TE = 60, 70, and 80 ms, were chosen to be averaged. **(B)** The averaged magnitude image. **(C)** The phase image of the last echo acquisition with TE of 80 ms. **(D)** The averaged magnitude image and the phase image of the last echo were applied to the susceptibility weighted imaging (SWI) processing to improve the signal-to-noise ratio (SNR) of the result. **(E)** The original SWI mlP result with a lower SNR.

**FIGURE 4:**
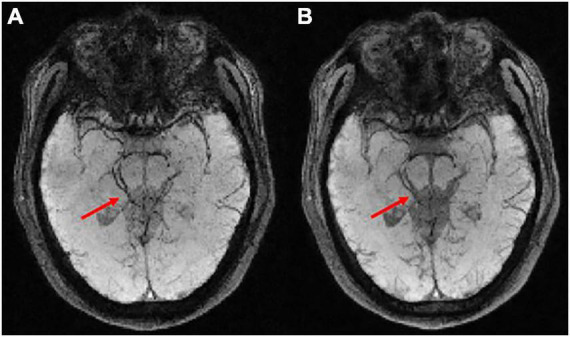
**(A)** The susceptibility weighted imaging (SWI) result was obtained through averaging the magnitude images of the last three echoes where TE = 60, 70, and 80 ms. **(B)** The SWI result was obtained through averaging the magnitude of all six echoes where TE = 30, 40, 50, 60, 70, and 80 ms.

Figure 3 shows the SWI results by averaging the magnitude images of the last three echoes to improve the SNR performance. As shown in Figures 3A,B, the averaged magnitude image has the similar contrast to the magnitude images of the last three echoes. The phase map from the last echo acquisition is shown in Figure 3C and is later combined with the averaged magnitude image for further SWI processing. The minimum intensity projection of SWI is shown in Figure 3D with a SNR of 46.3 compared to the original mIP image (Figure 3E) with a SNR of 39.3. Clearer structure of veins can be seen with this SNR improvement. Figure 4 compares the SWI results by averaging the magnitude images of the last three echoes and all six echoes. The more echoes used for averaging, the greater SNR improvement can be achieved. However, for the SWI result after averaging all six echoes, it is difficult to distinguish blood vessels from CSF as indicated by the red arrows, because the contrasts of the first three echoes may not satisfy the similar contrast requirements.

**FIGURE 5:**
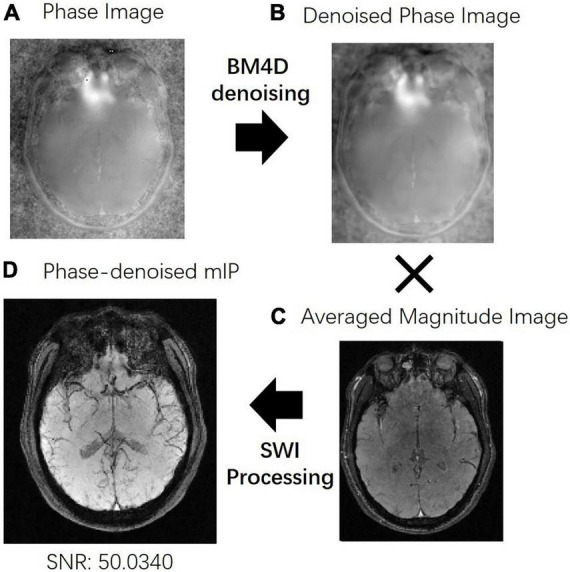
**(A)** The original phase image. **(B)** The original phase image was denoised using BM4D method to get the denoised phase image. **(C)** The average magnitude image of the last three echoes. **(D)** The averaged magnitude image and the denoised phase image were applied to the susceptibility weighted imaging (SWI) processing to improve the signal-to-noise ratio (SNR) of the result.

The results with a further SNR improvement using BM4D denoising are shown in Figure 5. The original phase image is quite noisy due to the long echo time acquisition with TE = 80 ms as shown in Figure 4A, while the denoised phase image keeps the fine phase information. It can be seen from Figure 5D that denoising can effectively improve the SNR, making the differences between different tissues larger, the target blood vessels clearer, and the background less noisy.

### 0.5T vs. 1.5T comparisons

**FIGURE 6:**
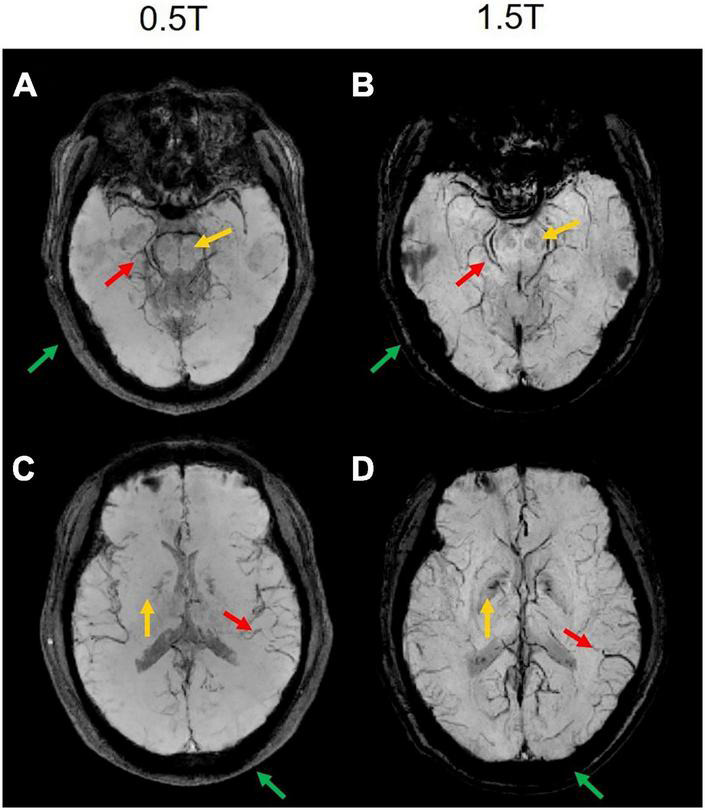
The comparison results of two representative slices from the same subject MRI scanning at 0.5T and 1.5T. **(A,C)** The magnitude-weighted susceptibility weighted images at 0.5T. **(B,D)** The magnitude-weighted susceptibility weighted images at 1.5T. Some small vessels are marked in red arrows, the red nucleus is marked in yellow arrows, the skull and extracranial lipid are marked in green arrows.

The comparison between the results from the 0.5T and 1.5T MRI is shown in Figure 6. It can be seen that high-field MRI can display some small blood vessels more clearly (red arrows in Figures 6B,D), while SWI images in 0.5T are also of good diagnostic value (red arrows in Figures 6A,C). Due to the different T2* decay at 0.5T and 1.5T, some signals at 0.5T decay more slowly than at 1.5T. For example, the skull and extracranial lipid are barely visible at 1.5T (green arrows in Figure 6), but is relatively clearer at 0.5T. Interesting, some details are difficult to identify at 0.5T compared to 1.5T, such as the red nucleus (yellow arrows in Figure 6).

## Discussion

In this work, we have shown that the protocol optimization and denoising method can improve SNR-starved SWI images acquired at low magnetic fields. We also proposed an innovative way to improve the image quality by averaging the magnitude images of several echoes, which takes full advantage of the relative flat T2* decay at low fields. Although some small blood vessels can be observed clearly in the 0.5T data after imaging protocol optimization and denoising, when the results at 0.5T are compared with those at 1.5T, the SNRs of the SWI images at 0.5T are relatively lower, and some tissues are indistinguishable. Furthermore, the clinical significance of the SWI images at 0.5T still needs further clinical verification.

In addition, in order to improve the scan efficiency, subsampling reconstruction techniques are expected to be applied to speed up sampling at low fields. While subsampling is very likely to further reduce the low SNR inherent in low-field MRI systems, we can improve it by using deep learning denoising techniques, which have provided high image quality sufficient for clinical applications at low fields in some studies (Zhu et al., 2018; Koonjoo et al., 2021). State-of-the-art denoising methods like BM4D were used in this work, which is based on nonlocal self-similarity image prior models, are popular and effective denoisers that generally act on the reconstructed magnitude images. However, these approaches typically employ time-consuming iterative optimizations that suffer from generalization to multiple noise levels. Deep learning can learn the noise and subtract it from the image to form its model, which needs less calculating time. What’s more, deep learning for subsampling strategy can also be considered for speeding up magnetic resonance imaging (Hyun et al., 2018; Bahadir et al., 2019). It is possible to develop more efficient and effective learning procedures for denoising or subsampling reconstruction for further improvement.

## Conclusion

In summary, this article optimized the imaging protocol to select suitable parameters such as the values of TE, TR, and the FA of the RF pulse according to the signal simulations for low-field SWI. Averaging multi-echo magnitude images and BM4D phase denoising were proposed to improve the SNR performance. From the SWI result obtained at 0.5T with the optimized parameters, we could identify the susceptibility difference between variable tissues and the target blood vessels clearly. When comparing the original image with the denoised image, significantly improved image SNR was observed. The SWI results at 0.5T demonstrated the capability to identify magnetic susceptibility differences between variable tissues, especially, the blood veins. This would open the possibility to use SWI in the high-performance low field MRI.

## Data availability statement

The raw data supporting the conclusions of this article will be made available by the authors, without undue reservation.

## Ethics statement

The studies involving human participants were reviewed and approved by the Institutional Review Board of Shanghai Jiao Tong University. The patients/participants provided their written informed consent to participate in this study.

## Author contributions

YQ, HC, and ZZ: conceptualization, methodology, and writing—original draft preparation. ZZ: project administration, funding acquisition, and supervision. YQ: formal analysis, validation, and investigation. YQ, HB, YZ, HL, and ZW: resources and data curation. YQ, HC, ZW, and ZZ: writing—review and editing. All authors contributed to the article and approved the submitted version.

## References

[B1] Bahadir C. D. Dalca A. V. Sabuncu M. R. (2019). Learning-based optimization of the under-sampling pattern in MRI. *Paper presented at the international conference on information processing in medical imaging*, (Hong Kong: Springer). 10.1002/hbm.24682

[B2] Barnes S. R. Haacke E. M. (2009). Susceptibility-weighted imaging: Clinical angiographic applications. *Magn. Reson. Imaging Clin. N. Am.* 17 47–61. 19364599 10.1016/j.mric.2008.12.002 PMC2713115

[B3] Bartzokis G. Sultzer D. Cummings J. Holt L. E. Hance D. B. Henderson V. W. (2000). *In vivo* evaluation of brain iron in Alzheimer disease using magnetic resonance imaging. *Arch. Gen. Psychiatry* 57 47–53. 10632232 10.1001/archpsyc.57.1.47

[B4] Bhat S. S. Fernandes T. T. Poojar P. da Silva Ferreira M. Rao P. C. Hanumantharaju M. C. (2021). Low-field MRI of stroke: Challenges and opportunities. *J. Magn. Reson. Imaging* 54 372–390. 10.1002/jmri.27324 32827173

[B5] Campbell-Washburn A. E. Ramasawmy R. Restivo M. C. Bhattacharya I. Basar B. Herzka D. A. (2019). Opportunities in interventional and diagnostic imaging by using high-performance low-field-strength MRI. *Radiology* 293 384–393. 10.1148/radiol.2019190452 31573398 PMC6823617

[B6] Coffey A. M. Truong M. L. Chekmenev E. Y. (2013). Low-field MRI can be more sensitive than high-field MRI. *J. Magn. Reson.* 237 169–174. 24239701 10.1016/j.jmr.2013.10.013 PMC3897717

[B7] Ejbjerg B. Narvestad E. Jacobsen S. Thomsen H. S. Østergaard M. (2005). Optimised, low cost, low field dedicated extremity MRI is highly specific and sensitive for synovitis and bone erosions in rheumatoid arthritis wrist and finger joints: Comparison with conventional high field MRI and radiography. *Ann. Rheum. Dis.* 64 1280–1287. 10.1136/ard.2004.029850 15650012 PMC1755626

[B8] Haacke E. M. Lai S. Reichenbach J. R. Kuppusamy K. Hoogenraad F. G. Takeichi H. (1997). *In vivo* measurement of blood oxygen saturation using magnetic resonance imaging: A direct validation of the blood oxygen level-dependent concept in functional brain imaging. *Hum. Brain Mapp.* 5 341–346. 10.1002/(SICI)1097-0193(1997)5:5<341::AID-HBM2>3.0.CO;2-3 20408238

[B9] Haacke E. M. Lai S. Yablonskiy D. A. Lin W. (1995). *In vivo* validation of the BOLD mechanism: A review of signal changes in gradient echo functional MRI in the presence of flow. *Int. J. Imaging Syst. Technol.* 6 153–163.

[B10] Haacke E. M. Mittal S. Wu Z. Neelavalli J. Cheng Y. C. (2009). Susceptibility-weighted imaging: Technical aspects and clinical applications, part 1. *AJNR Am. J. Neuroradiol.* 30 19–30. 19039041 10.3174/ajnr.A1400 PMC3805391

[B11] Haacke E. M. Xu Y. Cheng Y. C. Reichenbach J. R. (2004). Susceptibility weighted imaging (SWI). *Magn. Reson. Med.* 52 612–618. 15334582 10.1002/mrm.20198

[B12] Halefoglu A. M. Yousem D. M. (2018). Susceptibility weighted imaging: Clinical applications and future directions. *World J. Radiol.* 10:30. 10.4329/wjr.v10.i4.30 PMC5971274 29849962

[B13] Hori M. Hagiwara A. Goto M. Wada A. Aoki S. (2021). Low-field magnetic resonance imaging: Its history and renaissance. *Invest. Radiol.* 56:669. 10.1097/RLI.0000000000000810 34292257 PMC8505165

[B14] Hori M. Okubo T. Aoki S. Kumagai H. Araki T. (2006). Line scan diffusion tensor MRI at low magnetic field strength: Feasibility study of cervical spondylotic myelopathy in an early clinical stage. *J. Magn. Reson. Imaging* 23 183–188. 10.1002/jmri.20488 16374879

[B15] Hoult D. Chen C. N. Sank V. (1986). The field dependence of NMR imaging. II. Arguments concerning an optimal field strength. *Magn. Reson. Med.* 3 730–746. 10.1002/mrm.1910030509 3784890

[B16] Hyun C. M. Kim H. P. Lee S. M. Lee S. Seo J. K. (2018). Deep learning for undersampled MRI reconstruction. *Physics in Medicine Biology* 63 135007. 10.1088/1361-6560/aac71a 29787383

[B17] Jimeno M. M. Vaughan J. T. Geethanath S. (2022). Superconducting magnet designs and MRI accessibility: A review. *arXiv* [preprint] 10.48550/arXiv.2205.08918 36914280

[B18] Koonjoo N. Zhu B. Bagnall G. C. Bhutto D. Rosen M. S. (2021). Boosting the signal-to-noise of low-field MRI with deep learning image reconstruction. *Sci. Rep.* 11:8248. 10.1038/s41598-021-87482-7 33859218 PMC8050246

[B19] Liu C. Li W. Tong K. A. Yeom K. W. Kuzminski S. (2015). Susceptibility-weighted imaging and quantitative susceptibility mapping in the brain. *J. Magn. Reson. Imaging* 42 23–41. 25270052 10.1002/jmri.24768 PMC4406874

[B20] Marques J. P. van Kemenade W. Gazzo S. Grodzki D. Knopp E. A. Stainsby J. (2021). ESMRMB annual meeting roundtable discussion:“when less is more: The view of MRI vendors on low-field MRI”. *MAGMA* 34 479–482. 10.1007/s10334-021-00938-9 34259951 PMC8278376

[B21] Osmanodja F. Rösch J. Knott M. Doerfler A. Grodzki D. Uder M. (2022). Diagnostic performance of 0.55 T MRI for intracranial aneurysm detection. *Invest. Radiol*. 10.1097/RLI.0000000000000918 36070538

[B22] Peters A. M. Brookes M. J. Hoogenraad F. G. Gowland P. A. Francis S. T. Morris P. G. (2007). T2* measurements in human brain at 1.5, 3 and 7 T. *Magn. Reson. Imaging* 25 748–753. 10.1016/j.mri.2007.02.014 17459640

[B23] Reichenbach J. R. Venkatesan R. Schillinger D. J. Kido D. K. Haacke E. M. (1997). Small vessels in the human brain: MR venography with deoxyhemoglobin as an intrinsic contrast agent. *Radiology* 204 272–277. 9205259 10.1148/radiology.204.1.9205259

[B24] Runge V. M. Heverhagen J. T. (2022). The clinical utility of magnetic resonance imaging according to field strength, specifically addressing the breadth of current state-of-the-art systems, which include 0.55 T, 1.5 T, 3 T, and 7 T. *Invest. Radiol.* 57 1–12. 10.1097/RLI.0000000000000824 34510100

[B25] Saini S. Frankel R. B. Stark D. D. Ferrucci J. T. Jr. (1988). Magnetism: A primer and review. *AJR Am. J. Roentgenol.* 150 735–743. 3279729 10.2214/ajr.150.4.735

[B26] Santhosh K. Kesavadas C. Thomas B. Gupta A. K. Thamburaj K. Kapilamoorthy T. R. (2009). Susceptibility weighted imaging: A new tool in magnetic resonance imaging of stroke. *Clin. Radiol.* 64 74–83. 19070701 10.1016/j.crad.2008.04.022

[B27] Sarracanie M. Salameh N. (2020). Low-field MRI: How low can we go? A fresh view on an old debate. *Front. Phys.* 8:172. 10.3389/fphy.2020.00172

[B28] Schenck J. F. (1996). The role of magnetic susceptibility in magnetic resonance imaging: MRI magnetic compatibility of the first and second kinds. *Med. Phys.* 23 815–850. 10.1118/1.597854 8798169

[B29] Schweser F. Deistung A. Lehr B. W. Reichenbach J. R. (2010). Differentiation between diamagnetic and paramagnetic cerebral lesions based on magnetic susceptibility mapping. *Med. Phys.* 37 5165–5178. 10.1118/1.3481505 21089750

[B30] Sheth K. N. Mazurek M. H. Yuen M. M. Cahn B. A. Shah J. T. Ward A. (2021). Assessment of brain injury using portable, low-field magnetic resonance imaging at the bedside of critically ill patients. *JAMA Neurol.* 78 41–47. 10.1001/jamaneurol.2020.3263 PMC7489395 32897296

[B31] Stainsby J. A. Harris C. T. Bindseil G. A. Wiens C. N. Beatty P. J. Curtis A. T. (2019). “High-performance diffusion imaging on a 0.5 T system,” in *Proceedings of the 27th annual meeting of ISMRM*, Montreal, QC. 10.3390/polym11060941

[B32] Stainsby J. Bindseil G. A. Connell I. R. Thevathasan G. Curtis A. T. Beatty P. J. (2020). Imaging at 0.5 T with high-performance system components. *Proc. Intl. Soc. Mag. Reson. Med.* 27:1194. 10.3390/polym11060941 31151276 PMC6631666

[B33] Tong K. A. Ashwal S. Obenaus A. Nickerson J. P. Kido D. Haacke E. M. (2008). Susceptibility-weighted MR imaging: A review of clinical applications in children. *AJNR Am. J. Neuroradiol.* 29 9–17. 17925363 10.3174/ajnr.A0786 PMC8119104

[B34] Wang J. Mao W. Qiu M. Smith M. B. Constable R. T. (2006). Factors influencing flip angle mapping in MRI: RF pulse shape, slice-select gradients, off-resonance excitation, and B0 inhomogeneities. *Magn. Reson. Med.* 56 463–468. 10.1002/mrm.20947 16773653

[B35] Wang Y. Yu Y. Li D. Bae K. T. Brown J. J. Lin W. (2000). Artery and vein separation using susceptibility-dependent phase in contrast-enhanced MRA. *J. Magn. Reson. Imaging* 12 661–670. 10.1002/1522-2586(200011)12:5<661::aid-jmri2>3.0.co;2-l 11050635

[B36] Wiens C. N. Harris C. T. Curtis A. T. Beatty P. J. Stainsby J. A. (2020). *Feasibility of Diffusion Tensor Imaging at 0.5 T.* Concord, CA: ISMRM.

[B37] Wu Z. Mittal S. Kish K. Yu Y. Hu J. Haacke E. M. (2009). Identification of calcification with MRI using susceptibility-weighted imaging: A case study. *J. Magn. Reson. Imaging* 29 177–182. 19097156 10.1002/jmri.21617 PMC2646180

[B38] Wujciak D. (2022). Modern mid-field magnetic resonance imaging in private practice: Field report. *Radiologe* 62 405–409. 10.1007/s00117-022-00988-7 35482042

[B39] Zhu B. Liu J. Z. Cauley S. F. Rosen B. R. Rosen M. S. (2018). Image reconstruction by domain-transform manifold learning. *Nature* 555 487–492. 29565357 10.1038/nature25988

