# Supplementary material for: Susceptibility-weighted imaging at high-performance 0.5T magnetic resonance imaging system: Protocol considerations and experimental results
Source: Front Neurosci. 2022 Oct 12;16:999240. doi: 10.3389/fnins.2022.999240 (PMC9597077; doi:10.3389/fnins.2022.999240)
Supplement: Supplementary file 1 [file Image_1.pdf]

Supplemental information for

Susceptibility-weighted imaging at high-performance 0.5T MRI system: protocol considerations and experimental results

More volunteers

In Figure S1, five volunteer SWI images are shown. In these data we can see that blood vessels can be clearly displayed.

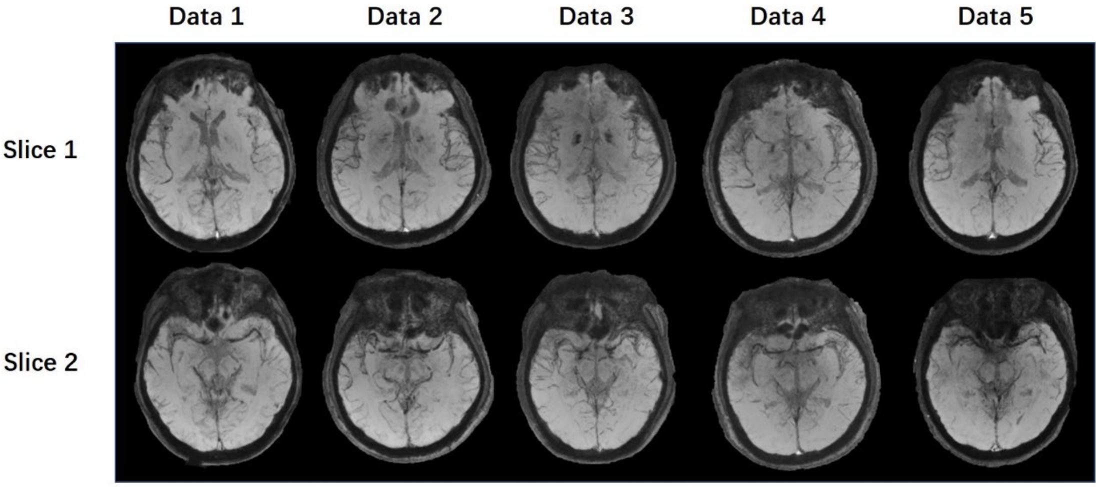

Figure S1 The figure shows the SWI images of five volunteers at 0.5T. The methods and parameters for obtaining images are the protocol proposed in our study. Different columns represent different volunteers, and different rows represent two layers with similar positions of different volunteers.
